# Characterization of the Mycoparasite *Cladosporium oxysporum* and Its Interaction with the Poplar Rust Pathogen *Melampsora larici-populina*

**DOI:** 10.3390/jof12040253

**Published:** 2026-04-01

**Authors:** Penghua Chen, Kuocheng Shen, Yadan Zhang, Zhongdong Yu, Qiangfeng Li

**Affiliations:** 1 College of Agriculture and Animal Husbandy, Qinghai University, Xining 810016, China; 18730872077@163.com; 2 State Key Laboratory of Forage Breeding-by-Design and Utilization, Institute of Botany, Chinese Academy of Sciences, Beijing 100093, China; 3 Longteng School, Longhua District Education Bureau, Shenzhen 518131, China; 4 College of Forestry, Northwest A&F University, Yangling 712100, China

**Keywords:** *Melampsora larici-populina*, mycoparasite, antifungal effects, *Cladosporium oxysporum*

## Abstract

Poplar rust, caused by *Melampsora larici-populina*, remains a primary biotic constraint limiting poplar growth. In this study, we characterized the diversity and antimicrobial potential of fungi associated with the aeciospore and urediniospore pustules of *M. larici-populina*. We isolated and identified 502 fungal strains, spanning 16 families, 22 genera, and 47 species. Comparative analysis revealed that the urediniospore stage harbored a greater abundance of fungal isolates but exhibited lower overall diversity, whereas the aeciospore stage was characterized by greater species richness despite lower individual strain counts. Notably, *Cladosporium oxysporum* was identified as the predominant species across both life stages, maintaining a consistent isolation frequency. We demonstrated that *C. oxysporum* exhibits strong antifungal activity; its metabolites contain bioactive components capable of degrading the cell walls of *M. larici-populina* urediniospores, resulting in an inhibition rate of 78.59%. Mechanistic observations via electron microscopy revealed that *C. oxysporum* develops appressoria to penetrate the urediniospore cell wall and subsequently forms haustoria-like structures within the protoplast to facilitate nutrient acquisition. This hyperparasitic interaction ultimately leads to the death of the rust pathogen. Collectively, our results identify *C. oxysporum* as a mycoparasite of *M. larici-populina* and highlight its potential as a promising biological control agent for the management of poplar rust.

## 1. Introduction

Since the outbreak of poplar rust in the 1990s, poplar rust disease has become a major constraint on poplar development, posing a significant threat to forestry production both in China and globally [1,2,3]. *Melampsora larici-populina*, one of the most widespread and destructive leaf rust pathogens, infects over 20 species of poplar, including sect. *Tacamahaca*, sect. *Aigeiros*, and their hybrids [4,5,6]. This disease primarily affects poplar leaves, causing them to lose their green color, form yellow spots, and eventually twist, curl, and die. This process leads to premature leaf drop, poor wood formation, and delayed bud break and leaf expansion the following year, significantly reducing tree growth and wood quality and causing substantial economic losses [7,8].

As a typical heteroecious, two-host rust fungus, *M. larici-populina* has a complex life cycle involving urediniospores, teliospores, basidiospores, spermatia, and aeciospores [9]. Diploid teliospores overwinter on senescent poplar leaves and produce haploid basidiospores in early spring. Wind-dispersed basidiospores infect larch needles, where they develop spermogonia that produce spermatia [10]. After fertilization, diploid aeciospores form and spread to poplar leaves, germinating to produce dikaryotic urediniospores. Urediniospores reproduce asexually, rapidly generating large quantities of pathogenic spores that infect nearby trees [11].

In the life cycle of *M. larici-populina*, the urediniospore stage represents the most destructive phase, characterized by recurrent infections on poplar leaves. During the asexual infection and proliferation process, urediniospores that attach to leaf surfaces germinate under humid conditions to form germ tubes [12]. These tubes primarily penetrate through stomata, differentiate into substomatal vesicles, and develop primary infection hyphae (also termed primary hyphae) within leaf tissues. Upon contact with mesophyll cells, a recognition process is initiated, leading to compatible interactions that induce the formation of haustorial mother cells. These structures pierce the host mesophyll cell walls to establish haustoria, specialized feeding organs that facilitate nutrient acquisition and sustain the parasitic relationship [13].

Current control strategies for *M. larici-populina* rely primarily on chemical fungicides and breeding resistant cultivars. However, chemical control can cause environmental pollution and ecosystem disruption, while resistant cultivar development faces challenges such as high costs, unstable genetic resistance, and technical complexities. Recently, biological control has gained attention, with increasing utilization of endophytes and mycoparasites. In particular, hyperparasitism—where obligate pathogens like powdery mildews, downy mildews, and rusts are parasitized by other fungi—has demonstrated great potential. Notable examples include *Tuberculino* and *Darluca filum*, which are widely applied in controlling blister rusts [14]. Recent evidence suggests that the genus *Cladosporium* may harbor widespread, yet underexploited, antagonistic potential against poplar rust. Specifically, *Cladosporium oxysporum* has recently been identified as a promising hyperparasite of *Melampsora medusae*, suggesting its broader utility as a novel biocontrol agent within these complex pathosystems [15,16].

Nevertheless, research on the diversity and mechanisms of mycoparasites infecting *M. larici-populina* remains limited. Therefore, this study aims to: (1) isolate and molecularly identify pustule-associated fungi from aeciospore and urediniospore stages of *M. larici-populina*; (2) analyze their diversity and dominant taxa; and (3) evaluate their antifungal effects and interaction mechanisms, thereby providing a foundation for biological control of *M. larici-populina*.

## 2. Materials and Methods

### 2.1. Experimental Materials

Pustule-associated fungi of poplar rust (*M. larici-populina*) were isolated from both the aeciospore and urediniospore stages. For the aeciospore stage, infected larch (*Larix* spp.) needles bearing aecial pustules were collected in May 2024 from the Huoditang Experimental Station, Shaanxi Province, China (34.31° N, 108.10° E, 435 m). For the urediniospore stage, infected poplar (*Populus cathayana*) leaves with uredinial pustules were collected in September 2024 from the same site. For each sampling group, specimens were obtained from at least 15 individual larch or poplar trees. From each tree, a minimum of 10 leaves infected by *M. larici-populina* and exhibiting fresh sporulating pustules were selected.

The rust pathogen was provided by the Forest Pathology Laboratory, Northwest A&F University, with accession number MLP0058. One-year-old *Populus purdomii* plants were used for antifungal assays.

### 2.2. Isolation of Pustule-Associated Fungi from M. larici-populina

To minimize the inclusion of fungi from the surrounding phyllosphere during isolation, we first selected intact infected samples in which the rust pustules showed no signs of physical compression or cross-contamination between leaves. Subsequently, the leaf surface within a 1 cm radius of the target rust pustules was thoroughly wiped three times with 95% ethanol to eliminate epiphytic contaminants. Finally, for infected samples, a direct spore isolation method was employed [15]. Aeciospores and urediniospores were aseptically scraped from pustules using a sterile needle under a stereomicroscope and cultured on Potato Dextrose Agar (PDA; 200 g potato, 20 g dextrose, 20 g agar, 1 L distilled water, pH adjusted to 7.0 with NaOH, autoclaved). At each stage, 120 pustules were randomly selected. Three pustules were inoculated per Petri dish, yielding a total of 80 plates. Cultures were incubated at 25 °C in darkness for 7 days. Emerging colonies were subcultured onto fresh PDA plates until pure isolates were obtained. Colony morphology and isolation frequency were documented.

### 2.3. DNA Extraction and PCR Amplification

Fungal isolates were initially grouped based on morphological characteristics, including colony appearance, pigmentation, hyphal structure, sporulation pattern, and spore morphology. For instance, isolates belonging to the genus *Cladosporium* were identified by their characteristic branched acropetal chains of conidia, where the youngest spores are situated at the apex and specialized ‘shield cells’ are frequently observed at branching points. Under an optical microscope, their conidiophores and conidia typically exhibit a pale to dark brown (dematiaceous) coloration, with distinctively thickened and darkened hila (attachment scars) at the spore ends. The conidia vary in shape from limoniform (lemon-shaped) to elliptical or cylindrical, with surfaces ranging from smooth to finely verrucose under high magnification. All morphological classifications and generic descriptions were verified with reference to Ainsworth & Bisby’s Dictionary of the Fungi. Microscopic observations were conducted after 10 days of growth at 25 °C. A total of 39 pustule-associated fungi from the aeciospore stage and 41 from the urediniospore stage were identified. Eighty representative isolates were selected for molecular identification based on internal transcribed spacer (ITS), partial actin (ACT), and the translation elongation factor 1-alpha (TEF1) gene sequences [15].

Genomic DNA was extracted using a CTAB protocol (65 °C lysis, chloroform: isoamyl alcohol purification, isopropanol precipitation) [13]. For PCR amplification, the ITS region was amplified using primers ITS1 (5′-TCCGTAGGTGAACCTGCGG-3′) and ITS4 (5′-TCCTCCGCTTATTGATATGC-3′). The PCR conditions were 94 °C for 5 min (initial denaturation), followed by 40 cycles of 94 °C (30 s), 54 °C (30 s), 72 °C (45 s), and a final extension at 72 °C for 10 min. ITS-based phylogenetic analysis was first used for species identification. Given the high isolation frequency and strong antifungal activity of *Cladosporium* species, additional amplification of ACT and TEF1 gene fragments was performed using primers ACT-512F/ACT-83R and EF1-728F/EF1-986R, respectively [17]. (ACT-512F: 5′-ATGTGCAAGGCCGGTTTCGC-3′; ACT-83R: 5′-TACGAGTCCTTCTGGCCAT-3; EF1-728F: 5′-CATCGAGAAGTTCGAGAAGG-3′; EF1-986R: 5′-TACTTGAAGGAACCCTTACC-3′). PCR products were sequenced (Sangon Biotech, Shanghai, China) and compared against the NCBI database using BLAST on line (https://blast.ncbi.nlm.nih.gov/Blast.cgi, accesed on 23 January 2026).

### 2.4. Phylogenetic Analysis

Homologous sequences were retrieved from NCBI, and multiple sequence alignment was performed using MEGA-X software (https://www.megasoftware.net/, accesed on 23 January 2026). A Neighbor-Joining tree was constructed based on the Maximum Composite Likelihood model with 1000 bootstrap replicates. Branch lengths represented evolutionary distances.

### 2.5. Diversity Analysis of Pustule-Associated Fungi

The relative isolation frequency (RIF) and tissue isolation frequency (TIF) of pustule-associated fungi were calculated at species, genus, family, and order levels for both aeciospore and urediniospore stages of *M. larici-populina*, using Formulas (1) and (2). RIF is defined as the isolation frequency of a specific fungal species relative to the total number of fungal isolates, whereas TIF indicates the average number of pustule-associated fungi that can be isolated from a single pustule [18].

(1) RIF = (Ni/Nt) × 100%

(2) TIF = (Nj/N) × 100%

Ni = The number of isolates of a specific species.

Nt = Total number of all isolates.

Nj = Isolation count of the jth pustule-associated fungi.

N = Total number of sampled pustules (aeciospore or urediniospore), with N = 120 in this study.

Species richness was evaluated by Margalef’s index (dMa) (Formula (3)), where higher dMa values indicate greater richness. Species diversity was evaluated by the Shannon-Wiener index (H) (Formula (4)) and Simpson’s index (λ) (Formula (5)). Increased H reflects higher diversity, whereas lower λ denotes greater diversity.

(3) dMa = (S − 1)/Ln (Nt)

S = Total number of pustule-associated fungi species identified.

(4) H = −1 × ∑(Nj/Nt × Ln (Nj/Nt))

(5) λ = ∑(Nj/Nt)^2^

Nt = Total number of pustule-associated fungi isolates.

Nj = Isolation count of the jth pustule-associated fungi species.

All calculations were performed separately for the aeciospore and urediniospore stages. These metrics collectively characterize the structural complexity of mycoparasitic communities across rust developmental stages.

### 2.6. Antifungal Assay

Healthy rust urediniospores were placed onto the surface of pustule-associated fungi cultured on PDA medium for 14 days. After incubation in darkness in Petri dishes for 24 h, spore germination and hyphal interactions were examined under a dissecting microscope. Temporary slides were prepared to observe spore deformation, lysis, and penetration by pustule-associated fungi using light microscopy.

### 2.7. Metabolite Assay of C. oxysporum

*C. oxysporum* was grown in potato dextrose broth (PD; 200 g potato, 20 g glucose, 1 L distilled water, pH adjusted to 7.0 with NaOH, autoclaved). Then, 100 mL conical flasks were incubated on a shaker (Innova 44, New Brunswick Scientific, Edison, NJ, USA) at 25 °C and 120 rpm for 10 days. Filter-sterilized metabolites were collected for bioassays. Urediniospores were treated with metabolites (200 µL) on concave slides. Germination rate and germ tube length were recorded after 24 h (humid chamber, darkness). For both the treatment and control groups, the germination rate and germ tube length of 10 randomly selected urediniospores were measured, with all experiments performed in triplicate. Data were analyzed using a two-tailed Student’s *t*-test. For in planta assays, *P. purdomii* leaves were sprayed with metabolites, then inoculated with urediniospores. Latent period and pustule density (day 11) were compared to PD controls. For both groups, the number of uredinia within a 1 cm^2^ square area on the leaves was counted. These experiments were conducted in triplicate, and statistical significance was determined using a two-tailed Student’s *t*-test.

### 2.8. Observation of Mycoparasitism by Scanning Electron Microscope

Urediniospores (1 × 106 spores/mL) were applied to *P. purdomii* leaves (100% RH, 25 °C, 24 h dark). Plants were transferred to a greenhouse (16 h light, 60–70% RH, 2500 lux). A spore suspension of *C. oxysporum* was sprayed on leaves 5 days post-rust inoculation sample. On day 15 after rust inoculation, diseased leaves were collected, and 0.5 × 0.5 cm samples were excised from affected areas while avoiding leaf veins. Samples were fixed in 4% glutaraldehyde solution for 8 h to preserve their native structure, then rinsed four times with 0.1 M phosphate buffer (pH 6.8) for 10 min each. Dehydration was performed sequentially in 30%, 50%, 70%, 80%, and 90% ethanol for 20 min each, followed by three 10 min incubations in 100% ethanol. Samples were subsequently exchanged once with isoamyl acetate for 20 min and dried in a CO_2_ critical point dryer. The dried specimens were mounted (abaxial side up) onto stubs using double-sided adhesive tape and coated with a ~20 nm platinum layer using a Hummer VI sputter coater. Interactions between the hyperparasite and the rust fungus were then examined and imaged with a Hitachi S-4800 scanning electron microscope (Hitachi High-Tech, Hitachinaka, Ibaraki, Japan [19].

For TEM processing, samples were fixed directly in Eppendorf vials with 3% glutaraldehyde in 0.1 M phosphate buffer (pH 7.2) for 18 h at 4×, dehydrated in a graded ethanol series, and embedded in LR WHITE acrylic resin (London Resin, London, UK) [17]. Thin sections (0.5–1 mm) were stained with 1% aq. toluidine blue and observed by means of LM. Ultrathin sections were collected on collodion-coated nickel grids, contrasted with uranyl acetate and lead citrate, and examined with a JEOL 100SX TEM (Jeol, Tokyo, Japan) operating at 80 kV.

## 3. Results

### 3.1. Diversity Analysis of Pustule-Associated Fungi Associated with M. larici-populina

We isolated a total of 502 fungal strains from the pustules of the poplar rust infection: 243 strains from the infected larch needles (aeciospore stage) and 259 strains from the infected poplar leaves (urediniospore stage). Morphological characterization initially identified 39 putative species from the aeciospore stage (designated MLPA-L1 to MLPA-L39) and 41 species from the urediniospore stage (MLPU-P1 to MLPU-P41), totaling 80 candidate pustule-associated fungi taxa.

DNA extraction and PCR amplification of the ITS region using primers ITS1/ITS4, followed by sequencing and NCBI BLAST analysis, classified the isolates into 16 distinct families (Figure S1). Phylogenetic analysis of each family (Figure S1) revealed that all isolates were clearly resolved at the genus level; Species-level identification was achieved for most taxa, except for those within *Alternaria*, which showed taxonomic complexity.

Final classification identified 47 species, 22 genera, and 16 families (Table 1). Taxonomic distribution showed that Ascomycota dominated (495 strains, 98.61%), comprising Dothideomycetes (66.93%), Sordariomycetes (30.28%), and Eurotiomycetes (1.39%). Basidiomycota accounted for only seven strains (1.40%), all belonging to Agaricomycetes (Table 1 and Table S1).

**Figure 1:**
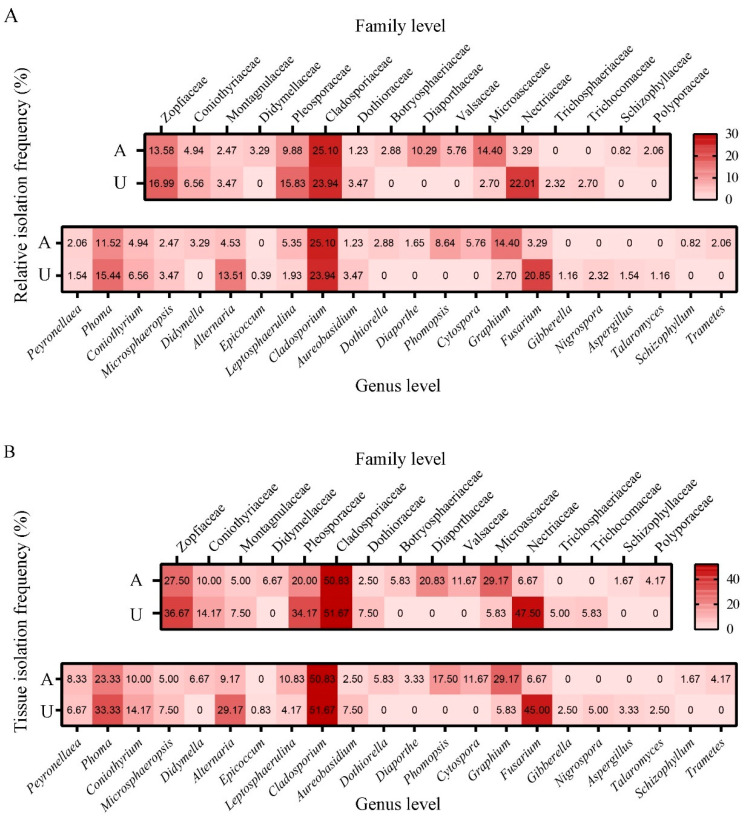
Relative isolation frequency (**A**) and tissue isolation frequency (**B**) of pustule-associated fungi at the level of family and genera in aeciospore and urediniospore stage of *M. larici-populina.* On the horizontal axis (x-axis), A represents aeciospore-associated fungi, and U represents urediniospore-associated fungi.

Community composition analysis revealed stage-specific differentiation of pustule-associated fungi. Aeciospore-associated communities comprised 14 families, 17 genera, and 29 species, whereas urediniospore-associated communities included 10 families, 15 genera, and 30 species (Figure 1; Table S1). Relative isolation frequency (RIF) analysis demonstrated that *Cladosporium* spp. served as the dominant taxon across both developmental stages. However, significant stage specificity was observed among secondary colonizers: members of *Microascaceae* (14.40%) and *Graphium* (14.40%) predominated in aeciospore communities, whereas *Nectriaceae* (22.01%) and *Fusarium* (20.85%) dominated urediniospore-associated assemblages (Figure 1A).

The tissue isolation frequency (TIF) quantitatively reflects the prevalence, colonization intensity, and isolation efficiency of pustule-associated fungi within rust pustules. Systematic analysis at family and genus levels (Figure 1B) revealed *Cladosporium* exhibited substantially higher TIF values than other pustule-associated fungi, with an average isolation rate of one strain per two rust pustules across both spore stages. The total TIF reached 205.5% in the aeciospore stage and 215.82% in the urediniospore stage, indicating that each rust pustule was, on average, colonized by approximately two fungal taxa.

Species richness and diversity analyses of pustule-associated fungi associated with *M. larici-populina* revealed distinct taxonomic partitioning between spore stages (Table S2). At the family level, urediniospore-stage communities lacked Didymellaceae, Dothioraceae, Diaporthaceae, and Valsaceae, whereas aeciospore-stage assemblages were missing only Gibberellaceae. Species-level analysis identified 18 and 17 stage-specific taxa for urediniospores and aeciospores respectively, demonstrating clear temporal selection. Urediniospore-associated fungi showed higher isolation frequency (259 vs. 243 strains) but lower richness, whereas aeciospore-stage communities exhibited greater taxonomic diversity despite reduced colonization density, as quantified by the Margalef (dMa) and Shannon (H) indices (Table S2).

### 3.2. Antifungal Efficacy of Pustule-Associated Fungi

**Figure 2:**
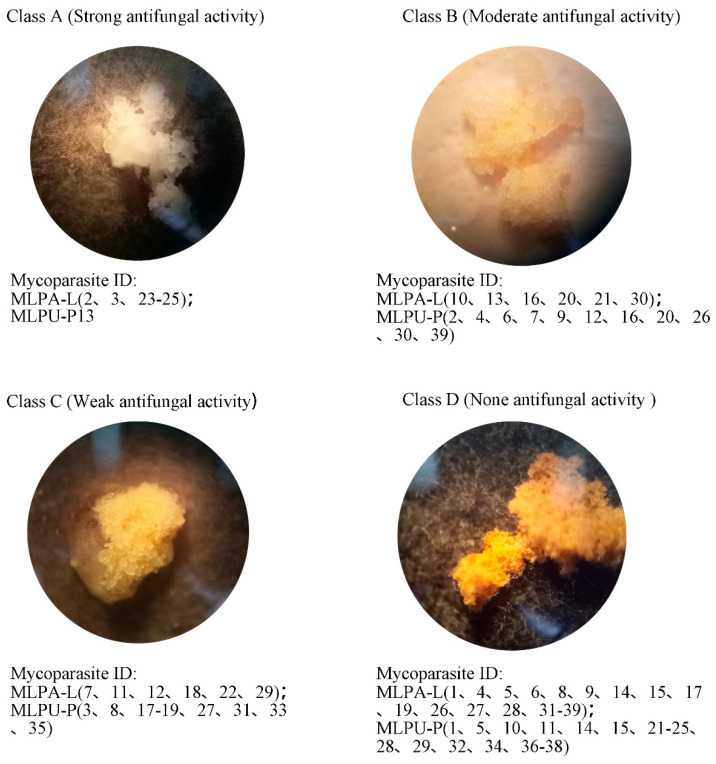
Antirust macro-activities of pustule-associated fungi from *M. larici–populina* at 24 h.

To evaluate antifungal activity, healthy urediniospore pustules were inoculated onto cultures of 80 pustule-associated fungi strains and observed after 24 h. The inhibitory effects were categorized into four classes (Figure 2). Class A (strong inhibition): Pustules turned completely white with crystalline-like liquefaction and were firmly adhered to the pustule-associated fungi. Microscopy revealed complete urediniospore whitening, cell wall lysis, and either protoplast formation or cytoplasmic leakage, with no germination.

Class B (moderate inhibition): Partial whitening without liquefaction. Urediniospores showed complete whitening but intact cell walls and no cytoplasmic leakage, with inhibited germination. Class C (weak inhibition): Light yellow discoloration without liquefaction. Microscopy detected partially whitened urediniospores with intact walls, mixed with germinated spores. Class D (no inhibition): Yellow pustules with visible germ tubes. Microscopy confirmed intact cell walls and widespread germination. These results suggest that effective fungal antagonists secrete metabolites with varying antifungal potency. In particular, Class A strains produced cell wall-lytic metabolites and showed tight physical association with urediniospores, indicating a potential capacity for direct penetration.

Quantitative isolation demonstrated that 117 (TIF = 97.5%) and 155 (TIF = 129.17%) inhibitory strains (class A–C) were recovered from aeciospore and urediniospore stages, respectively. This equates to approximately one fungal antagonist per wild-type pustule, underscoring their ecological significance in regulating rust growth and dissemination. Taxonomic analysis identified eight dominant genera with antifungal properties: *Cladosporium*, *Fusarium*, *Coniothyrium*, *Epicoccum*, *Phoma*, *Peyronellaea*, *Microsphaeropsis*, and *Gibberella*.

Notably, *Cladosporium* strains (Class A strong inhibitors) showed consistent prevalence across stages (aeciospores: TIF = 50.83%; urediniospores: TIF = 51.67%), suggesting stage-independent colonization stability. The >50% TIF values indicate *Cladosporium* spp. occurs in each two-pustule sampled, confirming its widespread distribution as a core fungal antagonist of *M. larici-populina*.

### 3.3. Antifungal Activity of C. oxysporum Metabolites

**Figure 3:**
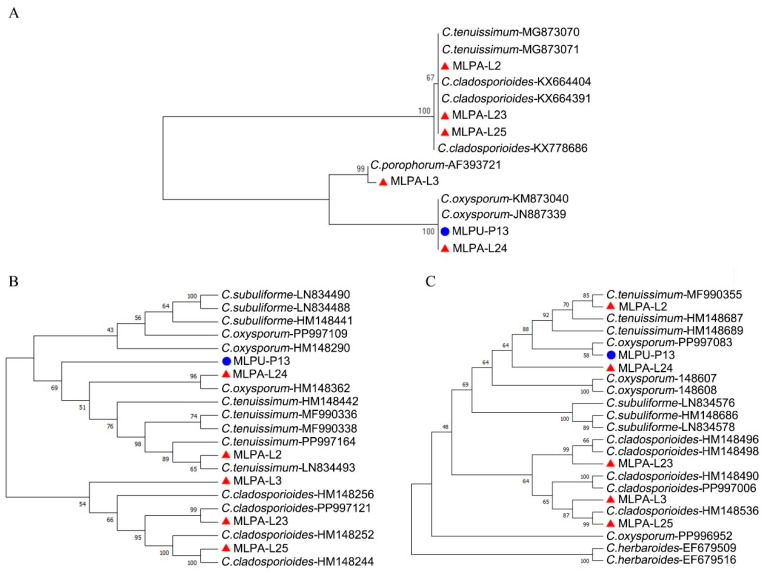
Phylogenetic trees of pustule-associated *Cladosporium*. The trees were constructed based on ITS (**A**), ACT (**B**), and TEF1 (**C**) gene fragment sequences. Red triangles represent aeciospore-associated fungi, and blue circles represent urediniospore-associated fungi.

We constructed a phylogenetic tree of the genus *Cladosporium* based on ITS sequences and found that *C. tenuissimum* and *C. cladosporioides* could not be reliably resolved at the species level (Figure 3A). By contrast, phylogenetic analyses based on partial actin (ACT) and translation elongation factor 1-alpha (TEF1) gene fragment sequences yielded a higher-resolution tree (Figure 3A,B). In this study, pustule-associated *Cladosporium* isolates were identified as *C. tenuissimum*, *C. cladosporioides*, and *C. oxysporum*. Among these, *C. oxysporum* was detected in both the aeciospore and urediniospore stages and exhibited the strongest antifungal activity (Figure 2).

**Figure 4:**
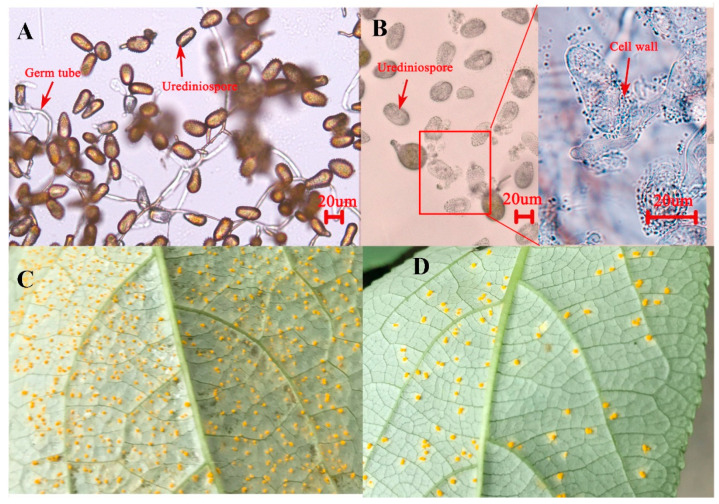
Anti-rust activities of metabolites from *C. oxysporum*. (**A**) Germination of *M. larici-populina* urediniospores in the control after 24 h of incubation. (**B**) Inhibition of *M. larici-populina* urediniospore germination by metabolites of *C. oxysporum* after 24 h of incubation. (**C**) Disease symptoms on *P. purdomii* leaves infected with *M. larici-populina* in the control at 11 days post-inoculation. (**D**) Suppression of *M. larici-populina* infection on *P. purdomii* leaves by *C. oxysporum* metabolites at 11 days post-inoculation.

Urediniospore germination assays with *C. oxysporum* metabolite extracts showed significant inhibition, with only 16.74 ± 4.33% germination compared to 73.92 ± 8.67% in controls (77.35% inhibition rate). Approximately 70% of treated spores exhibited complete whitening, with observable cell wall degradation and cytoplasmic leakage, suggesting direct lytic activity (Figure 4A,B).

In planta assays on *Populus purdomii* leaves demonstrated a marked reduction in disease severity, with treated leaves showing 18.47 ± 5.69 pustules cm^−2^ compared to 86.28 ± 7.52 pustules cm^−2^ in the control (78.59% suppression) (Figure 4C,D). The consistency between in vitro and in planta results confirms that metabolites of *C. oxysporum* contain potent antifungal compounds with cell wall-degrading activity, highlighting their strong potential for biological control of rust diseases. The combined evidence of spore lysis and in planta efficacy suggests that *C. oxysporum* represents a promising biocontrol agent against *M. larici-populina* infection.

### 3.4. C. oxysporum Is Identified as a Mycoparasite of M. larici-populina

**Figure 5:**
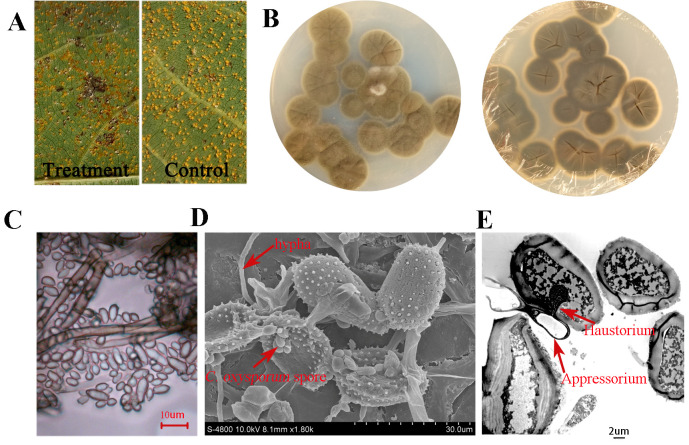
Interaction between *C. oxysporum* and *M. larici-populina*. (**A**) Antifungal effects following field application of a *C. oxysporum* spore suspension. Infected populus leaves were treated with a *C. oxysporum* spore suspension (treatment) or water (control) at 7 days post-inoculation with *M. larici-populina*. (**B**) Colony morphology of *C. oxysporum* after 7 days of growth on culture medium. (**C**) Conidial morphology of *C. oxysporum*. (**D**) Appressorium-like structures of *C. oxysporum* attached to urediniospores of *M. larici-populina*. Red arrows indicate the hyphae, and spore structures of *C. oxysporum*. (**E**) Penetration of urediniospore cell walls by *C. oxysporum* appressorium-like structures and formation of haustorium-like organs. Red arrows indicate appressoria and haustoria of *C. oxysporum*.

In field-based antifungal assays, leaf surface inoculation with *C. oxysporum* induced localized hypersensitive-like necrotic lesions accompanied by extensive whitening of rust pustules (Figure 5A). *C. oxysporum* exhibited typical morphological characteristics of the genus Cladosporium, with conidia approximately 5 μm in size (Figure 5B,C).

Scanning electron microscopy revealed dense colonization of uredinial pustules by *C. oxysporum* hyphae, accompanied by widespread collapse of urediniospores (Figure 5D). During mycoparasitism, abundant appressorium-like structures produced by *C. oxysporum* were observed to degrade the urediniospore cell wall and penetrate into the protoplast. Subsequently, *C. oxysporum* formed haustorium-like structures to extract nutrients, ultimately leading to the death of the rust fungus (Figure 5D,E).

This interaction, characterized by direct contact, host cell wall penetration, invasion of cellular contents, host cell death, and protoplast degradation, represents a typical mycoparasitic strategy. Collectively, these results demonstrate that *C. oxysporum* can be regarded as a mycoparasite of *M. larici-populina* with strong potential for biological control of poplar rust.

## 4. Discussion

In this study, we systematically investigated the diversity, antagonistic potential, and infection strategies of fungi associated with *Melampsora larici-populina*. Our results highlight both the ecological complexity of fungi communities colonizing rust pustules and the singular importance of the genus *Cladosporium*, particularly *C. oxysporum*, as a potential biocontrol agent.

Spore developmental stage significantly influenced both the composition and diversity of pustule-associated fungi. The aeciospore stage harbored a wider taxonomic spectrum, whereas the urediniospore stage supported denser colonization but lower overall diversity. This partitioning reflects ecological and physiological differences between the spore types: urediniospores are more ephemeral, rapidly produced propagules that generate abundant infection opportunities, thereby selecting for fast-growing fungi such as *Fusarium* and *Nectriaceae* members [20]. In contrast, aeciospores, as more durable structures, may provide longer-term niches that allow persistence of a broader taxonomic pool [18]. Similar spore stage-dependent structuring has been reported in other rust-associated communities, underscoring temporal niche partitioning as a driver of fungal diversity [21].

Among the diverse pustule-associated fungi recovered, *Cladosporium* emerged as the consistently dominant genus at both stages, accounting for more than half of the fungal isolates in certain pustules [1,15,16]. Such stability across developmental phases suggests ecological specialization and adaptation to rust microenvironments. Previous studies have documented *Cladosporium* spp. species as ubiquitous phyllosphere inhabitants with strong antagonistic activity towards rust fungi, particularly *Uromyces* and *Puccinia* species [19,20]. Our findings confirm and extend these reports, demonstrating that *C. oxysporum* metabolites not only suppress urediniospore germination in vitro but also achieve substantial disease reduction in planta. The dual evidence of spore lysis and leaf-level protection positions *C. oxysporum* as a promising candidate for field biocontrol applications.

The practical application of mycoparasites in forest pathology requires a thorough evaluation of host safety and potential phytotoxicity. In our field-based antifungal assays, we observed that leaf surface inoculation with *C. oxysporum* induced localized necrotic lesions. While these symptoms could be interpreted as opportunistic pathogenicity, the rapid whitening and collapse of *M. larici-populina* pustules suggest that these lesions may represent a localized hypersensitive response (HR), potentially triggered by the fungus as it targets the rust pathogen. Similar phenomena have been reported in other rust hyperparasites, where the biocontrol agent may elicit a transient defense response in the host plant while successfully colonizing the target pathogen [19,20]. Future research should prioritize systematic symptom scoring on healthy, non-infected leaves and formal Koch’s postulates through re-isolation to strictly define the ecological boundary between mycoparasitism and opportunistic infection [22]. To minimize ecological risks in practical forestry management, application strategies should be developed to maximize efficacy against rust while sparing host tissues.

Distinguishing between metabolite-mediated effects (antibiosis) and direct physical interaction (true mycoparasitism) is crucial for understanding the biocontrol potential of *C. oxysporum* [23,24]. Our findings demonstrate that this fungus employs a multifaceted strategy against *M. larici-populina* [3]. The antifungal activity observed in our crude extract assays (Figure 4) indicates a strong antibiosis component, likely driven by specialized secondary metabolites that inhibit spore germination. Simultaneously, the microscopic evidence of hyphal wrapping and penetration (Figure 5) confirms its role as a true mycoparasite. This dual mechanism aligns with previous reports of rust hyperparasites in forest systems, such as *Sphaerellopsis filum* or *Cladosporium* species, which often utilize enzymatic degradation to facilitate colonization [15,20].

While this study provides the most comprehensive overview to date of *M. larici-populina*-associated mycoparasites, several limitations remain. High-throughput sequencing approaches, such as amplicon-based metabarcoding, could be employed to complement culture-dependent methods and capture fastidious or unculturable taxa [22,25]. Moreover, chemical profiling of *C. oxysporum* metabolites will be critical to identify specific compounds responsible for antifungal activities and assess their potential as biopesticides. Finally, long-term field trials across diverse environments are necessary to validate the ecological stability and effectiveness of *C. oxysporum* -mediated biocontrol [18].

In summary, our study demonstrates that mycoparasitic fungi associated with *M. larici-populina* exhibit stage-specific diversity patterns, with *C. oxysporum* consistently emerging as the leading hyperparasite. By integrating ecological, microscopic, and metabolite evidence, we highlight that *C. oxysporum* is identified as a mycoparasite of *M. larici-populina*. These findings provide both theoretical and practical resources for advancing biological control strategies against poplar rust, contributing to sustainable forest disease management.

## 5. Conclusions

This study provides a comprehensive assessment of the diversity and biocontrol potential of pustule-associated fungi associated with the poplar rust pathogen, *M. larici-populina*. Our extensive isolation of 502 fungal strains reveals significant stage-specific variations in community structure: while the urediniospore stage supports a higher abundance of pustule-associated fungi, the aeciospore stage harbors greater species diversity. Despite these temporal variations, *C. oxysporum* emerged as the predominant and ecologically stable species across both developmental stages. Through in vitro and vivo assays, we demonstrated that *C. oxysporum* possesses potent antifungal activity; its metabolites contain bioactive components capable of degrading the cell walls of *M. larici-populina* urediniospores, achieving a 78.59% inhibition rate against poplar rust. Moreover, *C. oxysporum* was identified as a mycoparasite of *M. larici-populina*. During mycoparasitism, abundant appressorium-like structures produced by *C. oxysporum* degrade the urediniospore cell wall and penetrate into the protoplast. Subsequently, *C. oxysporum* formed haustorium-like structures to extract nutrients, ultimately leading to the death of the rust fungus. Collectively, these findings highlight the ecological complexity of rust-associated microbiomes and establish *C. oxysporum* as a promising, high-efficacy biological control agent for the sustainable management of poplar rust.

## Figures and Tables

**Table 1:** Summary of the pustule-associated fungi isolates from *M. larici-populina*.

Group	Morphotype	Taxa			
		Species	Family	Order (RIF)	Class (RIF)
A	MLPA-L (11, 18, 29); MLPU-P (31, 35)	*Peyronellaea prosopidis*	Zopfiaceae	Pleosporales (38.65%)	Dothideomycetes (66.93%)
	MLPA-L7	*Phoma aliena*			
	MLPA-L (10, 16); MLPU-P (2, 26, 30)	*Phoma moricola*			
	MLPU-P (9, 39)	*Phoma betae*			
B	MLPA-L (13, 30); MLPU-P4	*Coniothyrium aleuritis*	Coniothyriaceae		
	MLPU-P12	*Coniothyrium pyrinum*			
	MLPA-L20	*Coniothyrium* sp.			
C	MLPA-L12; MLPU-P (3, 27)	*Microsphaeropsis olivacea*	Montagnulaceae		
	MLPU-P33	*Microsphaeropsis amaranthi*			
D	MLPA-L (17, 19)	*Didymella glomerata*	Didymellaceae		
	MLPA-L8	*Didymella pomorum*			
E	MLPA-L27; MLPU-P (10, 22, 36, 40)	*Alternaria alternata*	Pleosporaceae		
	MLPU-P14	*Alternaria tenuissima*			
	MLPU-P (23, 37)	*Alternaria longipes*			
	MLPA-L1; MLPU-P38	*Alternaria brassicae*			
	MLPU-P41	*Alternaria yaliinficiens*			
	MLPU-P24	*Alternaria porri*			
	MLPU-P21	*Alternaria* sp.			
	MLPA-L21	*Epicoccum* sp.			
	MLPA-L9	*Leptosphaerulina arachidicola*			
	MLPA-L15; MLPU-P1	*Leptosphaerulina australis*			
	MLPA-L28; MLPU-P29	*Leptosphaerulina trifolii*			
F	MLPA-L2	*Cladosporium tenuissimum*	Cladosporiaceae	Capnodiales (24.50%)	
	MLPA-L (23, 25)	*Cladosporium cladosporioides*			
	MLPA-L3	*Cladosporium* *porophorum*			
	MLPA-L24; MLPU-P13	*Cladosporium* *oxysporum*			
G	MLPA-L (5, 6)	*Aureobasidium pullulans*	Dothioraceae	Dothideales (2.39%)	
H	MLPA-L31; MLPU-P (28, 32, 34)	*Dothiorella gregaria*	Botryosphaeriaceae	Botryosphaeriales (1.39%)	
I	MLPA-L (32, 34)	*Diaporthe nobilis*	Diaporthaceae	Diaporthales (7.77%)	Sordariomycetes (30.28%)
	MLPA-L33	*Phomopsis capsici*			
	MLPA-L35	*Phomopsis vaccinii*			
J	MLPA-L14	*Cytospora* sp.	Valsaceae		
K	MLPA-L4	*Graphium carbonarium*	Microascaceae	Microascales (9.96%)	
	MLPA-L (26, 38)	*Graphium euwallaceae*			
	MLPA-L39, 5	*Graphium penicillioides*			
L	MLPA-L22; MLPU-P18	*Fusarium fujikuroi*	Nectriaceae	Hypocreales (11.35%)	
	MLPU-P20	*Fusarium equiseti*			
	MLPU-P7	*Fusarium* sp.			
	MLPU-P19	*Fusarium oxysporum*			
	MLPU-P25	*Fusarium lateritium*			
	MLPU-P (6, 16)	*Gibberella fujikuroi*			
	MLPU-P (8, 17)	*Fusarium proliferatum*			
M	MLPU-P15	*Nigrospora aurantiaca*	Trichosphaeriaceae	Trichosphaeriales (1.20%)	
N	MLPU-P5	*Aspergillus japonicus*	Trichocomaceae	Eurotiales (1.39%)	Eurotiomycetes (1.39%)
	MLPU-P11	*Talaromyces pinophilus*			
O	MLPA-P36	*Schizophyllum commune*	Schizophyllaceae	Agaricales (0.40%)	Agaricomycetes (1.40%)
P	MLPA-P37	*Trametes hirsuta*	Polyporaceae	Polyporales (1.00%)	

## Data Availability

The original contributions presented in this study are included in the article/Supplementary Materials. Further inquiries can be directed to the corresponding authors.

## Supplementary Materials

The following supporting information can be downloaded at: https://www.mdpi.com/article/10.3390/jof12040253/s1, Figure S1. Phylogenetic relationships of *M. larici-populina*-associated fungi based on ITS sequences. Table S1. Isolation frequency of pustule-associated fungi of *M. larici-populina*. Table S2. Species richness and diversity analyses of pustule-associated fungi in aecional and uredinal stages from *M. larici-populina.*
