# Supplementary material for: Characterization of the Mycoparasite Cladosporium oxysporum and Its Interaction with the Poplar Rust Pathogen Melampsora larici-populina
Source: J Fungi (Basel). 2026 Apr 1;12(4):253. doi: 10.3390/jof12040253 (PMC13118109; doi:10.3390/jof12040253)
Supplement: Supplementary file 1 [file jof-12-00253-s001.zip › Supplementary-revised.pdf]

Supplementary information

Characterization of the Mycoparasite *Cladosporium oxysporum* and Its Interaction with the Poplar Rust Pathogen *Melampsora larici-populina*

Penghua Chen[1,†], Kuocheng Shen[2,†], Yadan Zhang[2,3,†],

Zhongdong Yu[4,*], Qiangfeng Li[1,*]

[1] College of Agriculture and Animal Husbandy, Qinghai University, Xining 810016, China

[2] State Key Laboratory of Forage Breeding-by-Design and Utilization, Institute of Botany, Chinese Academy of Sciences, Beijing, 100093, China

[3] Longteng School, Shenzhen, 518131 China.

[4] College of Forestry, Northwest A&F University, Yangling 712100, China

[†] These authors contributed equally to this work.

[*] Authors to whom correspondence should be addressed

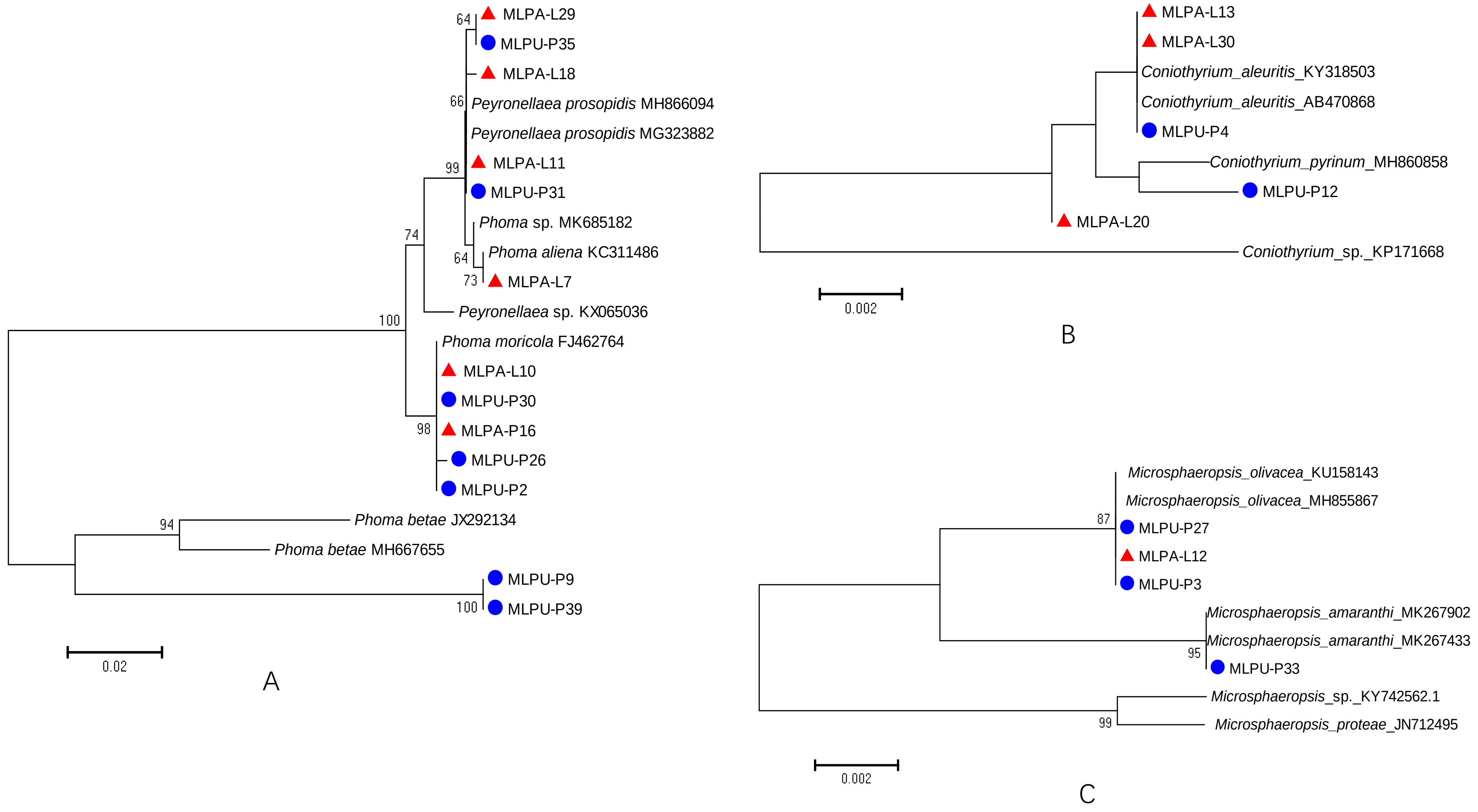

Figure S1. Phylogenetic relationships of *M. larici-populina*-associated fungi based on ITS sequences (A-C).

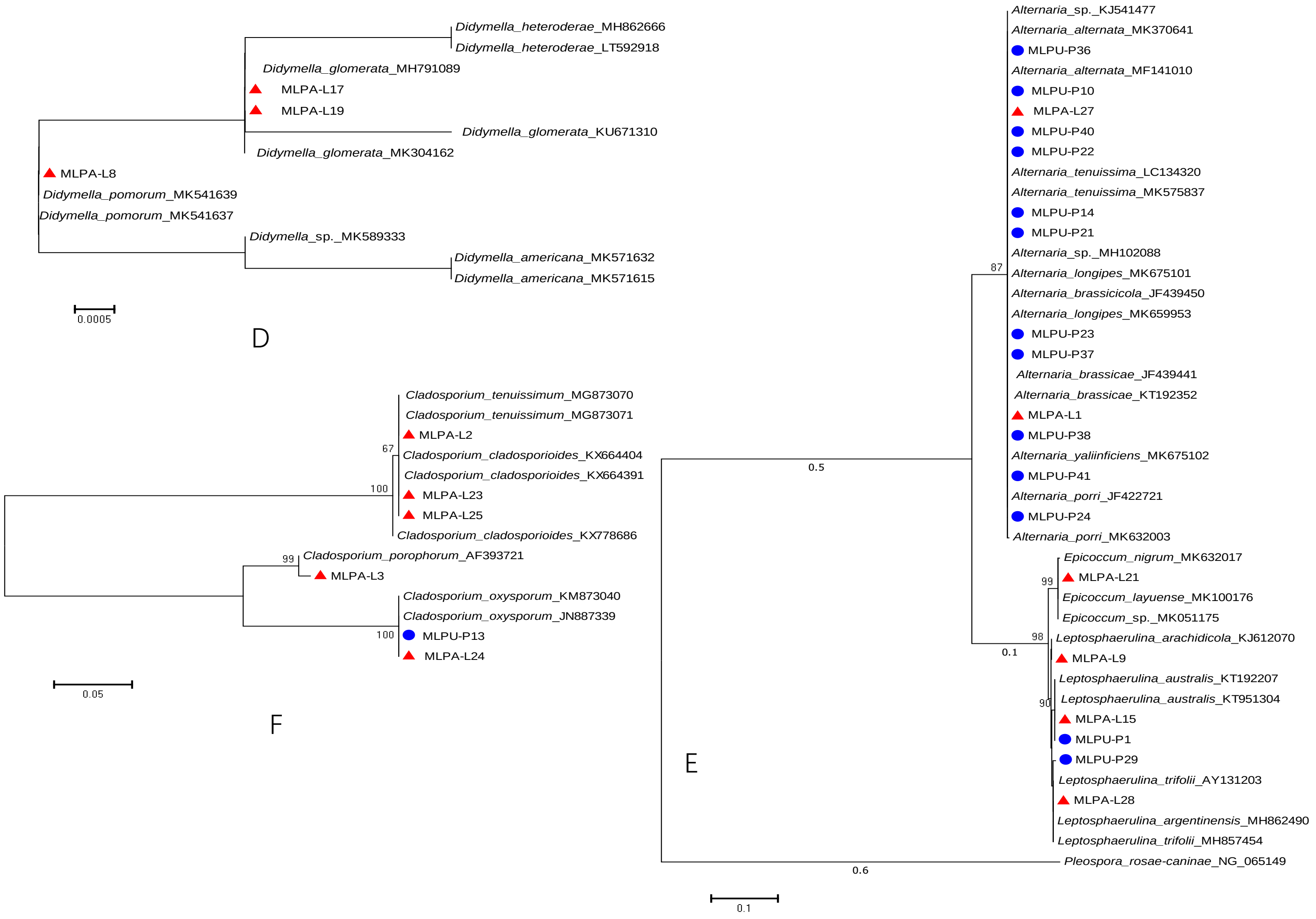

Figure S1. Phylogenetic relationships of *M. larici-populina*-associated fungi based on ITS sequences (D-F).

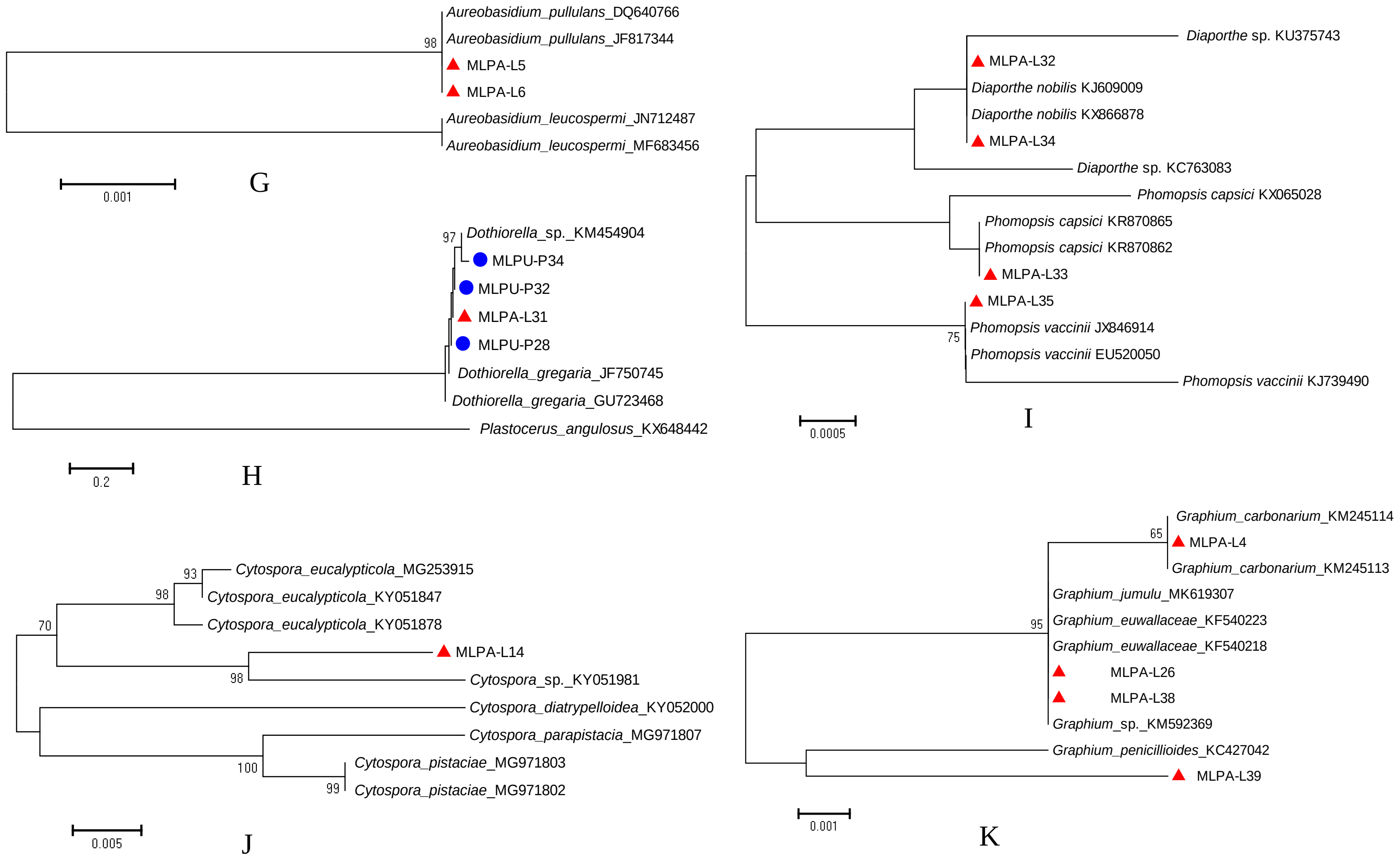

Figure S1. Phylogenetic relationships of *M. larici-populina*-associated fungi based on ITS sequences (G-K).

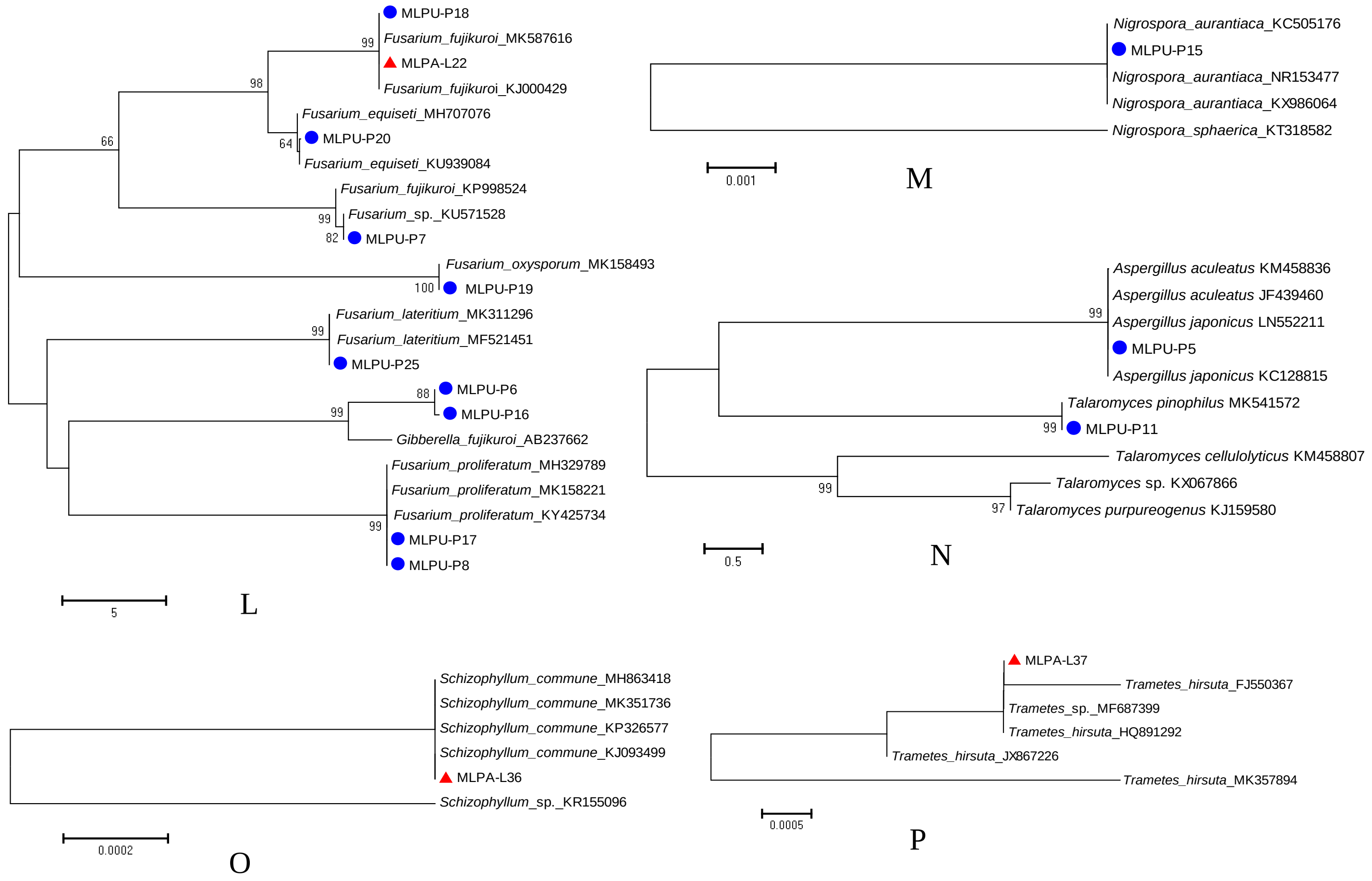

Figure S1. Phylogenetic relationships of *M. larici-populina*-associated fungi based on ITS sequences (L-P).

Maximum-likelihood analysis was performed using ITS rDNA sequences to resolve taxonomic placement of isolates. Symbols indicate host spore stage: red triangles indicate isolated from aeciospores; blue circles indicate isolated from urediniospores. Clades are annotated (A–P) to denote the 16 fungal families identified.

Table S1. Isolation frequency of mycoparasite of *M. larici –populina*

mycoparasite species	Aecional stage			Uredinal stage			All stage		
	N	RIF	TIF	N	RIF	TIF	N	RIF	TIF
Peyronellaea prosopidis	5	2.06	4.17	4	1.54	3.33	9	1.79	3.75
Phoma aliena	11	4.53	9.17	---	---	---	11	2.19	4.59
Phoma moricola	17	7.00	14.17	22	8.49	18.33	39	7.77	16.25
Phoma betae	---	---	---	18	6.95	15.00	18	3.59	7.50
Coniothyrium aleuritis	9	3.70	7.50	11	4.25	9.17	20	3.98	8.34
Coniothyrium pyrinum	---	---	---	6	2.32	5.00	6	1.20	2.50
Coniothyrium sp.	3	1.23	2.50	---	---	---	3	0.60	1.25
Microsphaeropsis olivacea	6	2.47	5.00	6	2.32	5.00	12	2.39	5.00
Microsphaeropsis amaranthi	---	---	---	3	1.16	2.50	3	0.60	1.25
Didymella glomerata	5	2.06	4.17	---	---	---	5	1.00	2.09
Didymella pomorum	3	1.23	2.5	---	---	---	3	0.60	1.25
Alternaria alternata	8	3.29	6.67	14	5.41	11.67	22	4.38	9.17
Alternaria tenuissima	---	---	---	2	0.77	1.67	2	0.40	0.84
Alternaria longipes	---	---	---	6	2.32	5.00	6	1.20	2.50
Alternaria brassicae	3	1.23	2.5	2	0.77	1.67	5	1.00	2.09
Alternaria yaliinficiens	---	---	---	5	1.93	4.17	5	1.00	2.09
Alternaria porri	---	---	---	2	0.77	1.67	2	0.40	0.84
Alternaria sp.	---	---	---	4	1.54	3.33	4	0.80	1.67
Epicoccum sp.	---	---	---	1	0.39	0.83	1	0.20	0.42
Leptosphaerulina arachidicola	6	2.47	5.00	---	---	---	6	1.20	2.50
Leptosphaerulina australis	2	0.82	1.67	---	---	---	2	0.40	0.84
Leptosphaerulina trifolii	5	2.06	4.17	5	1.93	4.17	10	1.99	4.17
Cladosporium tenuissimum	3	1.23	2.50	4	1.54	3.33	7	1.39	2.92
Cladosporium cladosporioides	11	4.53	9.17	---	---	---	11	2.19	4.59
Cladosporium porophorum	18	7.41	15.00	---	---	---	18	3.59	7.50
Cladosporium oxysporum	29	11.93	24.17	58	22.39	48.33	87	17.33	36.25
Aureobasidium pullulans	3	1.23	2.50	9	3.47	7.50	12	2.39	5.00
Dothiorella gregaria	7	2.88	5.83	---	---	---	7	1.39	2.92
Diaporthe nobilis	4	1.65	3.33	---	---	---	4	0.80	1.67
Phomopsis capsici	13	5.35	10.83	---	---	---	13	2.59	5.42

Phomopsis vaccinii	8	3.29	6.67	---	---	---	8	1.59	3.34
Cytospora sp.	14	5.76	11.67	---	---	---	14	2.79	5.84
Graphium carbonarium	18	7.41	15.00	---	---	---	18	3.59	7.50
Graphium euwallaceae	11	4.53	9.17	---	---	---	11	2.19	4.59
Graphium penicillioides	6	2.47	5.00	7	2.70	5.83	13	2.59	5.42
Fusarium fujikuroi	8	3.29	6.67	9	3.47	7.50	17	3.39	7.09
Fusarium equiseti	---	---	---	2	0.77	1.67	2	0.40	0.84
Fusarium sp.	---	---	---	8	3.09	6.67	8	1.59	3.34
Fusarium oxysporum	---	---	---	13	5.02	10.83	13	2.59	5.42
Fusarium lateritium	---	---	---	10	3.86	8.33	10	1.99	4.17
Gibberella fujikuroi	---	---	---	3	1.16	2.50	3	0.60	1.25
Fusarium proliferatum	---	---	---	12	4.63	10.00	12	2.39	5.00
Nigrospora aurantiaca	---	---	---	6	2.32	5.00	6	1.20	2.50
Aspergillus japonicus	---	---	---	4	1.54	3.33	4	0.80	1.67
Talaromyces pinophilus	---	---	---	3	1.16	2.50	3	0.60	1.25
Schizophyllum commune	2	0.82	1.67	---	---	---	2	0.40	0.84
Trametes hirsuta	5	2.06	4.17	---	---	---	5	1.00	2.09
Total	243	100	202.5	259	100	215.82	502	100	209.17

Table S2. Species richness and diversity analyses of pustule-associated fungi in aeciospore and urediniospore stage from *M. larici -populina*

Developmental stage	dMa	H	λ
Aeciospore stage	5.0973	3.1398	0.0526
Urediniospore stage	5.2188	2.9639	0.0816

Note:

The Margalef index (dMa) quantifies species richness, with higher values indicating greater richness.

The Shannon-Wiener index (H) and Simpson index (λ) measure diversity, where:

Higher H values indicate increased diversity,

Lower λ values reflect greater evenness and diversity.
